# Quality of ethnicity data within Scottish health records and implications of misclassification for ethnic inequalities in severe COVID-19: a national linked data study

**DOI:** 10.1093/pubmed/fdad196

**Published:** 2023-10-19

**Authors:** Sarah Amele, Ronan McCabe, Eliud Kibuchi, Anna Pearce, Kirsten Hainey, Evangelia Demou, Patricia Irizar, Dharmi Kapadia, Harry Taylor, James Nazroo, Laia Bécares, Duncan Buchanan, Paul Henery, Sandra Jayacodi, Lana Woolford, Colin R Simpson, Aziz Sheikh, Karen Jeffrey, Ting Shi, Luke Daines, Holly Tibble, Fatima Almaghrabi, Adeniyi Francis Fagbamigbe, Amanj Kurdi, Chris Robertson, Serena Pattaro, Srinivasa Vittal Katikireddi

**Affiliations:** MRC/CSO Social & Public Health Sciences Unit, University of Glasgow, Glasgow G12 8TB, UK; MRC/CSO Social & Public Health Sciences Unit, University of Glasgow, Glasgow G12 8TB, UK; MRC/CSO Social & Public Health Sciences Unit, University of Glasgow, Glasgow G12 8TB, UK; MRC/CSO Social & Public Health Sciences Unit, University of Glasgow, Glasgow G12 8TB, UK; MRC/CSO Social & Public Health Sciences Unit, University of Glasgow, Glasgow G12 8TB, UK; MRC/CSO Social & Public Health Sciences Unit, University of Glasgow, Glasgow G12 8TB, UK; Department of Sociology, School of Social Sciences, University of Manchester, Manchester M13 9PL, UK; Department of Sociology, School of Social Sciences, University of Manchester, Manchester M13 9PL, UK; Department of Sociology, School of Social Sciences, University of Manchester, Manchester M13 9PL, UK; Department of Sociology, School of Social Sciences, University of Manchester, Manchester M13 9PL, UK; Department of Global Health & Medicine, King's College London, London WC2B 4BG, UK; Research Data Scotland, Edinburgh EH8 9BT, UK; Public Health Scotland, Glasgow G2 6QE, UK; EAVE II Public Contributor; Usher Institute, University of Edinburgh, Edinburgh EH16 4SS, UK; Usher Institute, University of Edinburgh, Edinburgh EH16 4SS, UK; School of Health, Wellington Faculty of Health, Victoria University of Wellington, Wellington 6140, New Zealand; Usher Institute, University of Edinburgh, Edinburgh EH16 4SS, UK; Usher Institute, University of Edinburgh, Edinburgh EH16 4SS, UK; Usher Institute, University of Edinburgh, Edinburgh EH16 4SS, UK; Usher Institute, University of Edinburgh, Edinburgh EH16 4SS, UK; Usher Institute, University of Edinburgh, Edinburgh EH16 4SS, UK; Usher Institute, University of Edinburgh, Edinburgh EH16 4SS, UK; Institute of Applied Health Sciences, School of Medicine, Medical Sciences and Nutrition, University of Aberdeen, Aberdeen AB25 2ZD, UK; Strathclyde Institute of Pharmacy and Biomedical Sciences, University of Strathclyde, Glasgow G4 0RE, UK; Department of Pharmacology and Toxicology, College of Pharmacy, Hawler Medical University, Kurditsan Region Governorate, Erbil, Iraq; Department of Public Health Pharmacy and Management, School of Pharmacy, Sefako Makgatho Health Sciences University, Pretoria 0204, South Africa; Public Health Scotland, Glasgow G2 6QE, UK; Department of Mathematics and Statistics, University of Strathclyde, Glasgow G1 1XH, UK; Scottish Centre for Administrative Data Research, School of Social Political Sciences, University of Glasgow, Glasgow EH16 4UX, UK; MRC/CSO Social & Public Health Sciences Unit, University of Glasgow, Glasgow G12 8TB, UK

**Keywords:** quality, ethnicity, COVID-19

## Abstract

**Background:**

We compared the quality of ethnicity coding within the Public Health Scotland Ethnicity Look-up (PHS-EL) dataset, and other National Health Service datasets, with the 2011 Scottish Census.

**Methods:**

Measures of quality included the level of missingness and misclassification. We examined the impact of misclassification using Cox proportional hazards to compare the risk of severe coronavirus disease (COVID-19) (hospitalization & death) by ethnic group.

**Results:**

Misclassification within PHS-EL was higher for all minority ethnic groups [12.5 to 69.1%] compared with the White Scottish majority [5.1%] and highest in the White Gypsy/Traveller group [69.1%]. Missingness in PHS-EL was highest among the White Other British group [39%] and lowest among the Pakistani group [17%]. PHS-EL data often underestimated severe COVID-19 risk compared with Census data. e.g. in the White Gypsy/Traveller group the Hazard Ratio (HR) was 1.68 [95% Confidence Intervals (CI): 1.03, 2.74] compared with the White Scottish majority using Census ethnicity data and 0.73 [95% CI: 0.10, 5.15] using PHS-EL data; and HR was 2.03 [95% CI: 1.20, 3.44] in the Census for the Bangladeshi group versus 1.45 [95% CI: 0.75, 2.78] in PHS-EL.

**Conclusions:**

Poor quality ethnicity coding in health records can bias estimates, thereby threatening monitoring and understanding ethnic inequalities in health.

## Introduction

Ethnic inequalities in health have received heightened attention since the start of the coronavirus disease (COVID-19) pandemic, which has disproportionately impacted some minority ethnic groups.^1^^,^^2^ The availability of high-quality ethnicity data alongside health records is crucial to monitoring, understanding and redressing these inequalities.^3^ However, ethnicity data from UK health records have until recently been of limited use owing to poor completeness and quality.^4^

While there have been improvements, issues remain such as the inconsistent use of codes and high proportions of ‘not known’, ‘not stated’ or ‘other’ codes being used.^5^^,^^6^ For example, a study assessing the risk factors for severe COVID-19 early on in the pandemic in Scotland found that 15–26% of hospitalized individuals did not have their ethnicity recorded in NHS datasets.^7^ Such under-ascertainment can lead to the aggregation of heterogeneous ethnic groups, limited statistical power and the inability to effectively monitor ethnic inequalities in health.^7^

Ethnicity is a socially constructed, multi-dimensional and dynamic concept, where both the characteristics associated with ethnicity and individuals’ self-identification are fluid and context-dependent.^8–10^ Fixed-response categories commonly used to record ethnicity have been critiqued on these grounds, as has the aggregation of these categories in research.^8^^,^^11^ Nevertheless, fixed-response categories that are self-reported are generally viewed as preferable to those that are not (e.g. being ascribed by healthcare workers instead) for monitoring inequalities and quantitative research.^4^ As the Census in the UK adopts self-reported categories and offers almost complete population coverage, it is often considered the ‘gold standard’ and provides the best available ethnicity data.^12^ In contrast, health records are often incomplete and healthcare workers are not always aware that ethnicity should be self-reported.

This study aimed to (i) assess the quality of ethnicity coding within Scottish health datasets compared with the 2011 Scottish Census as the ‘gold standard’ and (ii) understand how differences in quality impact the observation of ethnic inequalities in severe COVID-19.

## Methods

### Study design

We used a cross-sectional analysis to assess the quality of ethnicity coding in Scottish health records compared with the ‘gold standard’ 2011 Scottish Census. We then used a population-based cohort analysis to explore the implications of misclassified ethnicity for assessing ethnic inequalities in severe COVID-19 (hospitalization or death).

### Study population and inclusion criteria

Individuals included were aged ≥16 years, present in both the Community Health Index (CHI) register and the 2011 Census, and residing in Scotland on 1 March 2020, the day of the first laboratory confirmed case of severe acute respiratory syndrome coronavirus 2 (SARS-CoV-2) in Scotland.^13^ CHI provides a unique numerical identifier for all registered patients in Scotland.

### Data

We used data from the Early Pandemic Evaluation and Enhanced Surveillance of COVID-19 (EAVE II) platform linked to data from the 2011 Scottish Census.^14^ The EAVE II study includes data for around 99% of the Scottish population (described in detail elsewhere).^15^ We used the following datasets: Public Health Scotland Ethnicity Look-up (PHS-EL), Electronic Communication or Surveillance in Scotland for SARS-CoV-2 testing data, Scottish Morbidity Record 01 (SMR-01) for hospitalizations, National Records of Scotland death registry (NRS deaths) and Accident & Emergency (A&E) services. PHS-EL was created during the pandemic to improve ethnicity information for monitoring and surveillance purposes. It includes the most recent ethnicity code from numerous National Health Service (NHS) Scotland datasets (Appendix 1), prior to 24 January 2022, and at the time of analysis represents the best available ethnicity information from NHS Scotland.

### Ethnicity

Ethnicity was classified using 16 categories from the 2011 Scottish Census, which were then aggregated to five categories for secondary analyses (Appendix 2); it should be noted, however, that the Caribbean and Black groups were available to us as a combined category despite existing in the Census as distinct categories.

Ethnicity data were complete in PHS-EL and the Census, with NRS having imputed for non-responses for the latter (2.1% for ethnic group in 2011).^16^ Cross-sectional analyses of SMR-01, NRS deaths and A&E were restricted to individuals with ethnicity codes in these datasets meeting the above inclusion criteria, as these datasets included ‘Missing’, ‘Unknown’ or ‘Not provided’ codes. The cohort analysis was restricted to individuals with ethnicity codes in PHS-EL meeting the inclusion criteria to provide a one-to-one comparison.

### Outcome

Our outcome for the cohort analysis was COVID-19 related hospitalization or death (referred to as severe COVID-19). The former was defined as a COVID-19 International Classification of Diseases (ICD) 10 code (U07.1-7) as the reason for admission (any position), or a reverse transcription polymerase chain reaction (RT-PCR) confirmed positive test for SARS-CoV-2 in the 28 days prior to admission. The latter was defined as either a death where the relevant ICD-10 code was recorded as the primary or secondary cause of death, or any death where the individual had a positive RT-PCR test for SARS-CoV-2 infection in the 28 days prior to death. Confirmed infection was defined as a positive RT-PCR laboratory test result. Severe COVID-19 was chosen as an exemplar outcome to understand the implications of misclassification. As such, this analysis did not provide any definite exploration of the relationship between ethnicity and severe COVID-19.

### Statistical analysis

Ethnicity coding in PHS-EL, SMR-01, NRS deaths and A&E were compared with the 2011 Scottish Census. We checked the level of misclassification in datasets compared with the Census. For the comparison with PHS-EL, we defined missingness as individuals present in the Census and CHI register without an ethnicity code in PHS-EL. We also calculated sensitivity and positive predictive value (PPV) for all comparisons in line with previous validation studies; sensitivity gave the proportion of individuals with a particular code in the Census who had a corresponding code in the comparator dataset, while PPV gave the proportion of individuals with a particular code in the comparator dataset who have a corresponding code in the Census.^17^^,^^18^ For the purposes of disclosure control, certain data from these analyses are withheld from publication to prevent differencing and low counts.

The cohort analysis used Cox proportional hazard models to estimate the risk of severe COVID-19 by ethnic group. All models were adjusted for age (5-year bands) and sex. Using calendar time, we followed individuals from 1 March 2020 (date of first SARS-CoV-2 case) until the first of: experiencing the outcome (hospitalization or death), death from any cause or 1 March 2022. We compared associations between models using ethnicity codes derived from the Census with models using coding from PHS-EL for these individuals. All analyses were conducted in the Scottish National Safe Haven using the R statistical software (Version 4.0.2).

### Patient and public involvement

Patient and public involvement (PPI) was carried out by the EAVE II PPI Coordinator and Public Advisory Group Co-Lead in collaboration with the study team (Appendix 3), helping with the prioritization of research questions and interpretation of findings.

## Results

### Cross-sectional analysis

A total of 3 776 564 unique individuals met the inclusion criteria for the analysis of PHS-EL. Socio-demographic characteristics for this population is presented in Appendix 4. Analyses of NRS deaths, SMR-01 and A&E concerned 141 726, 482 234 and 250 382 unique individuals, respectively. Owing to small numbers, socio-demographic characteristics for these populations are not presented.

#### Census versus PHS-EL

A total of 30% of individuals had missing ethnicity data in PHS-EL, this being highest among the White Other British group [39%] and lowest among the Pakistani group [17%] (Table 1). Overall, 8.5% of individuals in the Census were misclassified by PHS-EL (Table 1). Misclassification was highest for the White Gypsy/Traveller [69.1%; most often misclassified as White Scottish], Other Ethnicity [53.1%; most often misclassified as White Scottish] and Caribbean or Black [49.6%; most often misclassified as Mixed or Multiple Ethnicity] groups and lowest in the White Scottish group [5.1%] (Appendix 5 A and B). Sensitivity was highest for the Pakistani group [68.8%, 95% CI: 68.3 to 69.3] and lowest for the White Gypsy/Traveller group [3.9%, 95% CI: 3.2 to 4.7] (Appendix 6). PPV was high for the White Scottish [96.1%, 95% CI: 96.0 to 96.1], White Polish [94.6%, 95% CI: 94.3 to 95.0], Pakistani [93.2%, 95% CI: 92.9 to 93.5] and Chinese [92.6%, 95% CI: 92.0 to 93.1] groups (Appendix 6). PPV was low for the Other Ethnicity [9.6%, 95% CI: 8.9 to 10.4], White/Gypsy Traveller [28.3%, 95% CI: 23.9 to 33.1] and Mixed or Multiple Ethnicity [30.0%, 95% CI: 29.1 to 30.8] groups (Appendix 7).

**Table 1:** Comparison of dis-aggregated ethnicity coding within 2011 Census to Public Health Scotland ethnicity look-up variable

	Census	PHS-EL	Missing in PHS-EL	Misclassified in PHS-EL	Sensitivity	PPV
**Ethnicity**	***n* (%)**	***n* (%)**	**%**	**%**	**% (95% CI)**	**% (95% CI)**
White Scottish	3 265 450 (86.5)	2 206 598 (58.4)	30	5.1	64.9 (64.9, 65.0)	96.1 (96.0, 96.1)
White Other British	276 968 (7.3)	266 938 (7.1)	39	24.1	36.9 (36.7, 37.1)	38.3 (38.1, 38.5)
White Irish	36 007 (1.0)	10 591 (0.3)	34	44.1	21.9 (21.4, 22.3)	74.3 (73.5, 75.2)
White Gypsy/Traveller	2644 (0.1)	364 (0.0)	27	69.1	3.9 (3.2, 4.7)	28.3 (23.9, 33.1)
White Polish	35 300 (0.9)	14 047 (0.4)	22	40.3	37.7 (37.2, 38.2)	94.6 (94.3, 95.0)
Other White	53 404 (1.4)	57 828 (1.5)	28	25.6	46.4 (45.9, 46.8)	42.8 (42.4, 43.2)
Mixed or Multiple Ethnicity	10 197 (0.3)	11 296 (0.3)	23	43.8	33.2 (32.3, 34.1)	30.0 (29.1, 30.8)
Pakistani	30 812 (0.8)	22 737 (0.6)	17	14.2	68.8 (68.3, 69.3)	93.2 (92.9, 93.5)
Indian	16,380 (0.4)	12,132 (0.3)	23	16.6	60.4 (59.6, 61.1)	81.5 (80.8, 82.2)
Bangladeshi	2057 (0.1)	1419 (0.0)	25	20.7	54.3 (52.1, 56.4)	78.7 (76.5, 80.8)
Chinese	14 774 (0.4)	8382 (0.2)	35	12.5	52.5 (51.7, 53.3)	92.6 (92.0, 93.1)
Other Asian	10 787 (0.3)	7464 (0.2)	26	31.8	42.2 (41.3, 43.1)	61.0 (59.9, 62.1)
African	11 779 (0.3)	7778 (0.2)	25	21.1	53.9 (53.0, 54.8)	81.7 (80.8, 82.5)
Caribbean or Black	3016 (0.1)	2298 (0.1)	24	49.6	26.4 (24.9, 28.0)	34.7 (32.8, 36.7)
Arab	4181 (0.1)	1651 (0.0)	29	43.3	27.7 (26.3, 29.0)	70.1 (67.8, 72.2)
Other Ethnicity	2808 (0.1)	6105 (0.2)	26	53.1	20.9 (19.5, 22.5)	9.6 (8.9, 10.4)
Missing in PHS-EL	—	1 138 936 (30.2)	—	—	—	—
**Total**	3 776 564 (100.0)	3 776 564 (100.0)	30	8.5	—	—

As well as rounding the proportion (%) of missingness, the number of individuals missing and misclassified are not provided due to disclosure control.

When aggregated to five broad categories, missingness was > 22% for all groups (see Table 2). Misclassification was highest among the Mixed or Multiple Ethnicity group [44.2%] and lowest among the White group [0.3%], with these groups being most often misclassified as each other (Table 2 and Appendix 5–C). All other groups were most often misclassified as the White group. Sensitivity and PPV were highest for the White group [Sensitivity = 69.4, 95% CI: 69.3 to 69.4; PPV = 99.6, 95% CI: 99.6 to 99.6] and lowest for the Other Ethnicity group [Sensitivity = 29.8, 95% CI: 28.8 to 30.9; PPV = 26.9, 95% CI: 25.9 to 27.9] (Table 2).

**Table 2:** Comparison of aggregated ethnicity coding within 2011 Census to PHS ethnicity look-up variable

	Census	PHS-EL	Missing in PHS-EL	Misclassified in PHS-EL	Sensitivity	PPV
**Ethnicity aggregated**	***n*** **(%)**	***n*** **(%)**	***n*** **(%)**	***n*** **(%)**	**%** **(95% CI)**	**%** **(95% CI)**
White	3 669 773 (97.2)	2 556 366 (67.7)	1 113 623 (30.3)	9921 (0.3)	69.4 (69.3, 69.4)	99.6 (99.6, 99.6)
Mixed or Multiple Ethnicity	10 197 (0.3)	11 296 (0.3)	2299 (22.5)	4512 (44.2)	33.2 (32.3, 34.1)	30.0 (29.1, 30.8)
Asian	74 810 (2.0)	52 134 (1.4)	17 492 (23.4)	8473 (11.3)	65.3 (65.0, 65.6)	93.7 (93.5, 93.9)
African, Caribbean or Black	14 795 (0.4)	10 076 (0.3)	3599 (24.3)	2836 (19.2)	56.5 (55.7, 57.3)	83.0 (82.2, 83.7)
Other Ethnicity	6989 (0.2)	7756 (0.2)	1923 (27.5)	2982 (42.7)	29.8 (28.8, 30.9)	26.9 (25.9, 27.9)
Missing in PHS-EL	—	1,138,936 (30.2)	—	—	—	—
**Total**	3 776 564 (100.0)	3 776 564 (100.0)	1 138 936 (30.2)	28 724 (0.8)	—	—

#### Census versus SMR-01, census versus A&E and census versus NRS

When assessing disaggregated groups, sensitivity and PPV were highest among the White Scottish group for all comparisons (Appendices 6, 7 and 8A). The Mixed or Multiple Ethnicity group had the lowest sensitivity in the NRS deaths comparison [5.0%, 95% CI: 2.0 to 12.2], while the White Gypsy/Traveller group had the lowest sensitivity (<2.5%) in SMR-01 and A&E. The Mixed or Multiple Ethnicity group had the lowest PPV for both NRS deaths [9.8%, 95% CI: 3.9 to 22.5] and A&E [6.0%, 95% CI: 3.8 to 9.6] comparisons.

When assessing aggregated groups, misclassification was highest in the Mixed or Multiple Ethnicity group [*n* = 314; 71.0%; most often misclassified as White] and lowest in the White group [*n* = 589; 0.1%; most often misclassified as Other] in SMR-01 (Appendix 7–B). Sensitivity and PPV were highest among the White group and lowest among the Mixed or Multiple Ethnicity group (Appendices 6, 7, and 8B). While the proportion of ‘refused’ ethnicity codes in A&E was lowest among the Asian group [2.6%], this group had the second highest proportion of not known codes [15.1%] after the African, Caribbean or Black group [18.5%] (Appendix 8–C).

### Cohort analysis

**Fig. 1:**
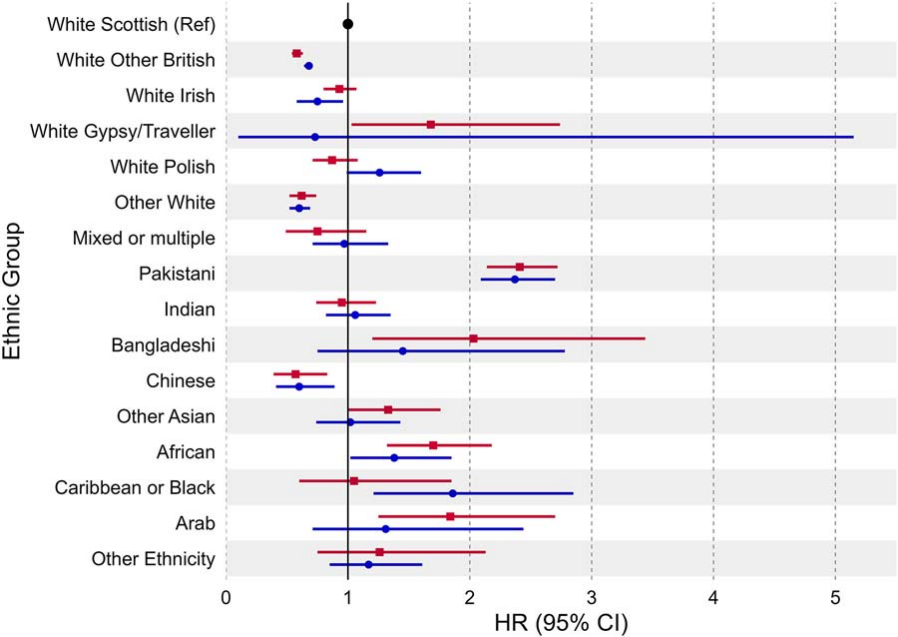
Risk of severe COVID-19 (hospitalization and death) using dis-aggregated ethnicity coding from the 2011 Scottish Census (first estimate in row) and from the Public Health Scotland ethnicity lookup (PHS-EL) dataset (second estimate in row).

**Fig. 2:**
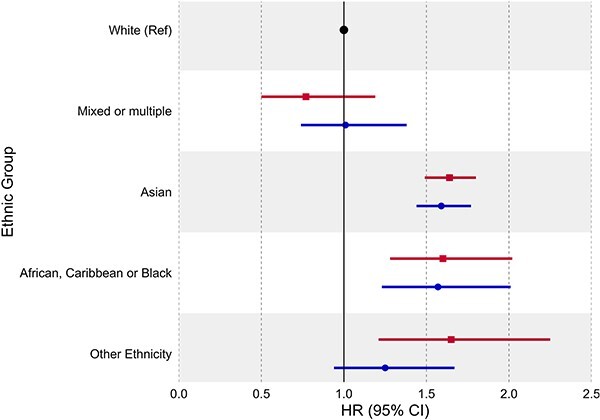
Risk of severe COVID-19 (hospitalization and death) using aggregated ethnicity coding from the 2011 Scottish Census (first estimate in row) and from the Public Health Scotland ethnicity lookup (PHS-EL) dataset (second estimate in row).

When using the Census ethnicity coding the White Gypsy/Traveller group had an elevated risk of severe COVID-19 [HR = 1.68, 95% CI: 1.03 to 2.74], however this was not observed when using PHS-EL [HR = 0.73, 95% CI: 0.10 to 5.15] (Appendix 9, Fig. 1). The Bangladeshi group exhibited an elevated risk using the Census coding [HR = 2.03, 95% CI: 1.20 to 3.44], but this was attenuated when using PHS-EL [HR = 1.45, 95% CI: 0.75 to 2.78]. Similar differences were observed for the Arab, African and Other Asian groups. Conversely, risk was elevated when using PHS-EL for the Caribbean or Black group [HR = 1.86, 95% CI: 1.21 to 2.85] compared to the Census [HR = 1.05, 95% CI: 0.60 to 1.85]. There were no major differences in risk for aggregated groups, with the exception of the Other Ethnicity group whose risk was attenuated when using PHS-EL [HR = 1.25, 95% CI: 0.94 to 1.67] compared to the Census [HR = 1.65, 95% CI: 1.21 to 2.25] (Appendix 9, Fig. 2).

## Discussion

### Main findings of this study

We examined the quality of ethnicity coding within four Scottish health datasets (PHS-EL, NRS, SMR-01 and A&E) compared with the 2011 Scottish Census. Using severe COVID-19 as an example, we highlighted the implications misclassification has for the monitoring of ethnic inequalities in health. For the main comparison between the 2011 Census and the best available ethnicity information from NHS Scotland (PHS-EL), we found that misclassification was lower in the White Scottish group [5.1%] compared to all other minority ethnic groups. Misclassification was notably high for the Caribbean or Black [49.6%] and the White Gypsy/Traveller [69.1%] groups. Sensitivity was highest for the Pakistani group [68.8%] and lowest for the White Gypsy/Traveller group [3.9%]. PPV was also low for the White Gypsy/Traveller group. We showed that misclassification in PHS-EL conceals the high risk of severe COVID-19 among the White Gypsy/Traveller group and underestimates risks for the Bangladeshi, Arab and Caribbean and Black groups. When conducting analyses using broad aggregated ethnic groups, misclassification was highest for the Mixed and Multiple Ethnicity group and lowest for the White group, with missingness > 22% across all groups.

### What is already known on this topic?

Previous validation studies have similarly found the quality of ethnicity data to be worse among minority ethnic groups compared to the White majority and notable inconsistencies for the Mixed or Multiple ethnicity group.^18–20^ It should be noted, however, that more recent validation studies have focused on aggregated ethnic groups only.^19^^,^^20^ Relatedly, studies and surveillance reports rarely reported dis-aggregated ethnicity data early in the COVID-19 pandemic.^21^ Aggregating groups can conceal important heterogeneity between ethnic groups^11^; we show that aggregated groups conceal the elevated level of misclassification and risk of severe COVID-19 among the White Gypsy/Traveller group, as the aggregated White group is dominated by the White Scottish majority.

There has also been a lack of recent validity studies both across the UK and within Scotland.^17^^,^^20^^,^^22^ One previous study evaluated the accuracy of a name-based classification system using ethnicity coding from Scottish administrative datasets; however, the quality of coding was not considered, the datasets used were only regional and did not contain data on health outcomes, thus offering limited insight into the implications that ethnicity data quality has for the observation of health inequalities.^17^

### What this study adds

We provide a population-level evaluation of the quality of ethnicity data in Scottish health datasets and clearly demonstrate how both the quality and granularity of ethnicity coding influences the observation of ethnic inequalities in health. In particular, we clearly demonstrate this to be the case for the White Gypsy/Traveller group, which is important as sizeable health inequalities exist between Gypsy/Traveller and non-Gypsy/Traveller populations in the UK.^23^ Additionally, we are the first to show that this minority ethnic group is at an increased risk of severe COVID-19 in Scotland. More generally, we highlight that data linkage may offer improved ethnicity data quality. This approach differs, for example, from addressing data quality issues using name-based classification systems which are often of questionable validity, not least for individuals of Mixed or Multiple ethnicity. ^4^^,^^17^^,^^22^^,^^24^

### Limitations of this study

We treat the 2011 Census as the ‘gold standard’ by which the quality of the other datasets is assessed. However, as ethnicity is labile, it is possible that for certain individuals PHS-EL (and other datasets) may represent a more up-to-date representation of an individual’s ethnicity. A study in England and Wales found self-reported ethnicity to be stable for 96% of individuals between the 2001 and 2011 Censuses, although stability was lower among ethnic minority groups.^25^ Relatedly, it is possible that some individuals may have mistakenly provided the wrong ethnicity in the Census. Additionally, it is also worth considering that, for the cohort analysis, estimates for certain groups were derived from low counts of both individuals and events. Lastly, we did not examine whether individuals present in the CHI register or PHS-EL but not the Census (who were excluded) differed in their demographic characteristics from those included in our analyses.

## Supplementary Material

QualityofEthnicityData_suppmaterial_fdad196

## Data Availability

The data used in this study include sensitive category individual-level data. To prevent disclosure these data are not publicly available but are available for research purposes through successful application to NHS Scotland Public Benefit and Privacy Panel for Health and Social Care (HSC-PBPP) and National Records of Scotland (NRS).

## Notes

**Sarah Amele**, Dr

**Ronan McCabe**, Dr

**Eliud Kibuchi**, Dr

**Anna Pearce**, Dr

**Kirsten Hainey**, Dr

**Evangelia Demou**, Dr

**Patricia Irizar**, Dr

**Dharmi Kapadia**, Dr

**Harry Taylor**, Dr

**James Nazroo**, Professor of Sociology

**Laia Bécares**, Professor of Social Science and Health

**Duncan Buchanan**, Dr

**Paul Henery**, Dr

**Sandra Jayacodi**,

**Lana Woolford**, Dr

**Colin R Simpson**, Proffessor of population health

**Aziz Sheikh**, Professor of Primary Care Research and Development, Director of the Usher Institute

**Karen Jeffrey**, Dr

**Ting Shi**, Dr

**Luke Daines**, Dr

**Holly Tibble**, Dr

**Fatima Almaghrabi**, Dr

**Adeniyi Francis Fagbamigbe**, Dr

**Amanj Kurdi**, Dr

**Chris Robertson**, Professor of Public Health Epidemiology

**Serena Pattaro**, Dr

**Srinivasa Vittal Katikireddi**, Professor of Public Health and Health Inequalities
