# Supplementary material for: Quality of ethnicity data within Scottish health records and implications of misclassification for ethnic inequalities in severe COVID-19: a national linked data study
Source: J Public Health (Oxf). 2023 Oct 19;46(1):116–22. doi: 10.1093/pubmed/fdad196 (PMC10901260; doi:10.1093/pubmed/fdad196)
Supplement: QualityofEthnicityData_suppmaterial_fdad196 [file qualityofethnicitydata_suppmaterial_fdad196.docx]

# APPENDICES

***Appendix 1***

PHS-EL draws data from the following NHS Scotland datasets: Scottish Morbidity Record (SMR) 01, SMR00, Rapid Preliminary Inpatient Data (RAPID), Electronic Communication of Surveillance in Scotland (ECOSS) and Case Management System and Unscheduled Care

***Appendix 2***

Sixteen (dis-aggregated) and five (aggregated) ethnicity categories used in the analyses. For disaggregated category 14, Caribbean and Black classifications are presented separately in the Census (indicated by ‘/') but were provided to us as an aggregated category.

| ***Dis-aggregated categories*** | ***Aggregated categories*** |
| --- | --- |
| 1. White Scottish | 1. White |
| 2. White Other British |  |
| 3. White Irish |  |
| 4. White Gypsy/Traveller |  |
| 5. White Polish |  |
| 6. Other White |  |
| 7. Mixed or Multiple Ethnicity | 1. Mixed or Multiple Ethnicity |
| 8. Pakistani, Pakistani Scottish or Pakistani British (referred to in main text as Pakistani) | 1. Asian |
| 9. Indian, Indian Scottish or Indian British (referred to in main text as Indian) |  |
| 10. Bangladeshi, Bangladeshi Scottish or Bangladeshi British (referred to in main text as Bangladeshi) |  |
| 11. Chinese, Chinese Scottish or Chinese British (referred to in main text as Chinese) |  |
| 12. Other Asian |  |
| 13. African, African Scottish or African British (referred to in main text as African) | 1. African, Caribbean or Black |
| 14. Caribbean, Caribbean Scottish or Caribbean British/Black, Black Scottish or Black British (referred to in main text as African or Black) |  |
| 15. Arab, Arab Scottish or Arab British (referred to in main text as Arab) | 1. Other Ethnicity |
| 16. Other Ethnicity |  |

***Appendix 3***

During analysis design refinement, the EAVE II PAG (n=9) were invited to discuss ethnicity classifications, aggregation, and other data considerations with staff. The PPI team were invited to attend project meetings, discuss results with staff and contribute to this manuscript.

***Appendix 4***

Demographic characteristics of individuals present in both the Census and the CHI register, with a known ethnicity in the former. These individuals formed the study population for the main comparison between the Census and the PHS Ethnicity Look-up (PHS-EL) dataset.

|  | **Level** | **N** | **%** |
| --- | --- | --- | --- |
| **Total** |  | 3776564 | 100.0 |
| Sex | Male | 1808876 | 47.9 |
|  | Female | 1967688 | 52.1 |
| Age (years) | Mean (SD) | 50.9 (19.4) | n/a |
|  | Median (IQR) | 52 (66-35) | n/a |
|  | 16-64 | 2768095 | 73.3 |
|  | 65-79 | 731182 | 19.4 |
|  | >79 | 277287 | 7.3 |
| NHS Health board | Ayrshire & Arran | 274848 | 7.3 |
|  | Borders | 78842 | 2.1 |
|  | Dumfries & Galloway | 108825 | 2.9 |
|  | Forth Valley | 203490 | 5.4 |
|  | Grampian | 408297 | 10.8 |
|  | Highland | 218699 | 5.8 |
|  | Lothian | 576535 | 15.3 |
|  | Orkney | 15059 | 0.4 |
|  | Shetland | 16065 | 0.4 |
|  | Western Isles | 18680 | 0.5 |
|  | Fife | 253631 | 6.7 |
|  | Tayside | 284924 | 7.5 |
|  | Greater Glasgow & Clyde | 807639 | 21.4 |
|  | Lanarkshire | 465303 | 12.3 |
|  | *Missing* | 45727 | 1.2 |
| SIMD | One (highest) | 718406 | 19.0 |
|  | Two | 748964 | 19.8 |
|  | Three | 757143 | 20.0 |
|  | Four | 761895 | 20.2 |
|  | Five (lowest) | 744429 | 19.7 |
|  | *Missing* | 45727 | 1.2 |
| Urban-rural | 1. Large urban areas | 1227464 | 32.5 |
|  | 2. Other urban areas | 1365844 | 36.2 |
|  | 3. Accessible small towns | 325795 | 8.6 |
|  | 4. Remote small towns | 85673 | 2.3 |
|  | 5. Very remote small towns | 47839 | 1.3 |
|  | 6. Accessible rural areas | 428230 | 11.3 |
|  | 7. Remote rural areas | 114758 | 3.0 |
|  | 8. Very remote rural areas | 101503 | 2.7 |
|  | *Missing* | 79458 | 2.1 |

***Appendix 5***

A. Cross table of dis-aggregated groups for the main comparison between the Census and PHS-EL. Correct classifications are shaded in blue. This table only compares the White groups and combines the White Polish and Other White groups owing low numbers and thus is not a complete cross table of dis-aggregated groups.

|  |  | **PHS-EL** | | | | |
| --- | --- | --- | --- | --- | --- | --- |
|  | **Ethnic group** | White Scottish | White Other British | White Irish | White Gypsy/Traveller | White Polish/Other White |
| **Census** | White Scottish | 2119367 | 155429 | 1089 | 210 | 13965 |
|  | White Other British | 59490 | 102204 | 1481 | 27 | 5169 |
|  | White Irish | 12372 | 3075 | 7873 | 10 | 391 |
|  | White Gypsy/Traveller | 1436 | 231 | 10 | 103 | 138 |
|  | White Polish/Other White | 7666 | 4017 | 107 | 11 | 50358 |

B. Cross table of dis-aggregated groups for the main comparison between the Census and PHS-EL. Correct classifications are shaded in blue. This table excludes certain groups and combines others (African and Caribbean and Black) owing to low numbers and thus is not a complete cross table of dis-aggregated groups.

|  |  | **PHS-EL** | | | | | | | | |
| --- | --- | --- | --- | --- | --- | --- | --- | --- | --- | --- |
|  | **Ethnic group** | White Scottish | White Other British | Mixed or multiple ethnic groups | Pakistani | Indian | Other Asian | African/ Caribbean or Black | Arab | Other ethnic group |
| **Census** | White Scottish | 2119367 | 155429 | 2408 | 321 | 250 | 375 | 417 | 103 | 937 |
|  | White Other British | 59490 | 102204 | 444 | 54 | 40 | 113 | 126 | 23 | 285 |
|  | Mixed or multiple ethnic groups | 2085 | 600 | 3386 | 102 | 112 | 233 | 252 | 32 | 299 |
|  | Pakistani, Pakistani Scottish or Pakistani British | 1103 | 272 | 817 | 21191 | 874 | 643 | 99 | 34 | 293 |
|  | Indian, Indian Scottish or Indian British | 590 | 215 | 540 | 417 | 9893 | 516 | 86 | 11 | 218 |
|  | Other Asian | 414 | 170 | 589 | 339 | 553 | 4552 | 78 | 46 | 788 |
|  | African/Caribbean or Black | 812 | 291 | 925 | 38 | 57 | 77 | 8360 | 85 | 299 |
|  | Arab, Arab Scottish or Arab British | 379 | 119 | 355 | 70 | 51 | 222 | 228 | 1157 | 255 |
|  | Other ethnic group | 275 | 120 | 234 | 67 | 140 | 184 | 81 | 84 | 588 |

C. Cross table of aggregated groups for the main comparison between the Census and PHS-EL. Correct classifications are shaded in blue.

|  |  | **PHS-EL** | | | | | | **Total** |
| --- | --- | --- | --- | --- | --- | --- | --- | --- |
|  | **Ethnic group** | White | Mixed or multiple | Asian | African, Caribbean or Black | Other Ethnic Group | **Missing** |  |
| **Census** | White | 2546229 | 3962 | 1772 | 855 | 3332 | 1113623 | 3669773 |
|  | Mixed or multiple ethnic groups | 3340 | 3386 | 589 | 252 | 331 | 2299 | 10197 |
|  | Asian | 4114 | 2434 | 48845 | 300 | 1625 | 17492 | 74810 |
|  | African, Caribbean or Black | 1348 | 925 | 179 | 8360 | 384 | 3599 | 14795 |
|  | Other Ethnic Group | 1335 | 589 | 749 | 309 | 2084 | 1923 | 6989 |
| **Total** | | 2556366 | 11296 | 52134 | 10076 | 7756 | 1138936 | 3776564 |

***Appendix 6***

A. Sensitivity and PPV for comparison between the Census and NRS Deaths using dis-aggregated groups.

|  | **Sensitivity** | **PPV** |
| --- | --- | --- |
| **Ethnicity** | Estimate (95% CI) | Estimate (95% CI) |
| White Scottish | 95.0 (94.9, 95.2) | 98.1 (98.0, 98.2) |
| White Other British | 81.1 (80.3, 82.0) | 55.6 (54.6, 56.5) |
| White Irish | 46.1 (43.3, 48.9) | 73.3 (70.0, 76.3) |
| White Gypsy/Traveller | 9.5 (4.7, 18.3) | 58.3 (32.0, 80.7) |
| White Polish | 82.2 (75.2, 87.5) | 91.6 (85.6, 95.2) |
| Other White | 59.1 (55.0, 63.2) | 28.5 (26.0, 31.2) |
| Mixed or multiple ethnic groups | 5.0 (2.0, 12.2) | 9.8 (3.9, 22.5) |
| Pakistani, Pakistani Scottish or Pakistani British | 68.5 (63.7, 72.9) | 95.0 (91.8, 97.0) |
| Indian, Indian Scottish or Indian British | 67.6 (59.5, 74.8) | 67.6 (59.5, 74.8) |
| Bangladeshi, Bangladeshi Scottish or Bangladeshi British | 44.4 (24.6, 66.3) | 88.9 (56.5, 98.0) |
| Chinese, Chinese Scottish or Chinese British | 66.3 (58.7, 73.1) | 95.6 (90.1, 98.1) |
| Other Asian | 30.5 (20.3, 43.1) | 36.0 (24.1, 49.9) |
| African | 35.4 (23.4, 49.6) | 39.5 (26.4, 54.4) |
| Caribbean or Black | 28.3 (17.3, 42.5) | 50.0 (32.1, 67.9) |
| Arab, Arab Scottish or Arab British | 17.4 (7.0, 37.1) | 66.7 (30.0, 90.3) |
| Other ethnic group | 8.7 (2.4, 26.8) | 10.5 (2.9, 31.4) |

B. Sensitivity and PPV for comparison between the Census and NRS Deaths using aggregated groups.

|  | **Sensitivity** | **PPV** |
| --- | --- | --- |
| **Ethnicity** | Estimate (95% CI) | Estimate (95% CI) |
| White | 99.9 (99.9, 99.9) | 99.7 (99.7, 99.7) |
| Mixed or multiple ethnic groups | 5.0 (2.0, 12.2) | 9.8 (3.9, 22.5) |
| Asian | 72.1 (68.8, 75.1) | 93.6 (91.3, 95.3) |
| African, Caribbean or Black | 39.4 (30.1, 49.5) | 53.6 (42.0, 64.9) |
| Other ethnic group | 17.4 (9.1, 30.7) | 32.0 (17.2, 51.6) |

***Appendix 7***

A. Sensitivity and PPV for comparison between the Census and SMR-01 using dis-aggregated groups.

|  | **Sensitivity** | **PPV** |
| --- | --- | --- |
| **Ethnicity** | Estimate (95% CI) | Estimate (95% CI) |
| White Scottish | 78.1 (78.0, 78.2) | 96.0 (95.9, 96.1) |
| White Other British | 48.8 (48.2, 49.4) | 31.6 (31.2, 32.0) |
| White Irish | 22.7 (21.4, 24.1) | 70.0 (67.4, 72.5) |
| White Gypsy/Traveller | 2.3 (1.2, 4.5) | 44.4 (24.6, 66.3) |
| White Polish | 60.4 (57.7, 63.1) | 93.1 (91.1, 94.6) |
| Other White | 33.8 (32.1, 35.5) | 26.3 (24.9, 27.7) |
| Mixed or multiple ethnic groups | 10.6 (8.1, 13.9) | 11.1 (8.4, 14.4) |
| Pakistani, Pakistani Scottish or Pakistani British | 61.2 (59.2, 63.1) | 91.2 (89.7, 92.5) |
| Indian, Indian Scottish or Indian British | 49.6 (46.5, 52.8) | 68.9 (65.3, 72.2) |
| Bangladeshi, Bangladeshi Scottish or Bangladeshi British | 44.8 (36.6, 53.2) | 65.2 (55.1, 74.2) |
| Chinese, Chinese Scottish or Chinese British | 58.4 (54.4, 62.2) | 91.5 (88.3, 93.9) |
| Other Asian | 26.1 (22.4, 30.2) | 37.8 (32.8, 43.1) |
| African | 39.4 (35.3, 43.6) | 67.2 (61.8, 72.2) |
| Caribbean or Black | 28.7 (23.1, 35.1) | 32.0 (25.8, 38.8) |
| Arab, Arab Scottish or Arab British | 13.7 (9.9, 18.6) | 62.3 (48.8, 74.1) |
| Other ethnic group | 14.8 (10.3, 20.8) | 5.1 (3.5, 7.3) |

B. Sensitivity and PPV for comparison between the Census and SMR-01 using aggregated groups.

|  | **Sensitivity** | **PPV** |
| --- | --- | --- |
| **Ethnicity** | Estimate (95% CI) | Estimate (95% CI) |
| White | 86.0 (85.9, 86.1) | 99.7 (99.7, 99.7) |
| Mixed or multiple ethnic groups | 10.6 (8.1, 13.9) | 11.1 (8.4, 14.4) |
| Asian | 63.6 (62.2, 65.0) | 93.7 (92.8, 94.5) |
| African, Caribbean or Black | 47.9 (44.4, 51.5) | 70.9 (66.8, 74.7) |
| Other ethnic group | 21.8 (18.1, 26.0) | 16.1 (13.3, 19.3) |

C. N (%) of composition, refused, not known and misclassified for comparison between the Census and SMR-01 using aggregated groups. Note that the column total for SMR-01 includes Refused and Not Known, and column percentages are derived from this total.

|  | **Census** | **SMR-01** | **Refused** | **Not Known** | **Misclassified** |
| --- | --- | --- | --- | --- | --- |
| **Ethnicity aggregated** | **n (%)** | **n (%)** | **n (%)** | **n (%)** | **n (%)** |
| White | 403742 (98.5) | 348419 (85.0) | 17196 (4.3) | 38611 (9.6) | 589 (0.1) |
| Mixed or Multiple Ethnicity | 442 (0.1) | 424 (0.1) | 30 (6.8) | 51 (11.5) | 314 (71.0) |
| Asian | 4561 (1.1) | 3099 (0.8) | 234 (5.1) | 557 (12.2) | 867 (19.0) |
| African, Caribbean or Black | 747 (0.2) | 505 (0.1) | 46 (6.2) | 98 (13.1) | 245 (32.8) |
| Other Ethnicity | 417 (0.1) | 566 (0.1) | 29 (7.0) | 44 (10.6) | 253 (60.7) |
| **Total** | 409,909 (100.0) | 409,909 (100.0) | 17535 (4.3) | 39361 (9.6) | 2268 (0.6) |

D. Cross table of aggregated groups for the main comparison between the Census and SMR-01. Correct classifications are shaded in blue.

|  |  | **SMR-01** | | | | | | | **Total** |
| --- | --- | --- | --- | --- | --- | --- | --- | --- | --- |
|  | **Ethnic group** | White | Mixed or multiple | Asian | African, Caribbean or Black | Other Ethnic Group | Refused | Not Known |  |
| **Census** | White | 347346 | 127 | 96 | 80 | 286 | 17196 | 38611 | 403742 |
|  | Mixed or multiple ethnic groups | 260 | 47 | 23 | 18 | 13 | 30 | 51 | 442 |
|  | Asian | 504 | 182 | 2903 | 33 | 148 | 234 | 557 | 4561 |
|  | African, Caribbean or Black | 168 | 31 | 18 | 358 | 28 | 46 | 98 | 747 |
|  | Other Ethnic Group | 141 | 37 | 59 | 16 | 91 | 29 | 44 | 417 |
| **Total** | | 348419 | 424 | 3099 | 505 | 566 | 17535 | 39361 | 409909 |

***Appendix 8***

A. Sensitivity and PPV for comparison between the Census and A&E using dis-aggregated groups.

|  | **Sensitivity** | **PPV** |
| --- | --- | --- |
| **Ethnicity** | Estimate (95% CI) | Estimate (95% CI) |
| White Scottish | 79.3 (79.1, 79.5) | 96.0 (95.9, 96.1) |
| White Other British | 46.0 (45.1, 46.8) | 28.2 (27.6, 28.8) |
| White Irish | 20.3 (18.7, 22.1) | 69.4 (65.7, 72.8) |
| White Gypsy/Traveller | 2.2 (0.8, 5.4) | 44.4 (18.9, 73.3) |
| White Polish | 64.0 (60.4, 67.5) | 95.0 (92.6, 96.6) |
| Other White | 30.1 (27.8, 32.5) | 35.4 (32.8, 38.1) |
| Mixed or multiple ethnic groups | 7.1 (4.4, 11.3) | 6.0 (3.8, 9.6) |
| Pakistani, Pakistani Scottish or Pakistani British | 60.7 (58.1, 63.3) | 91.1 (89.1, 92.8) |
| Indian, Indian Scottish or Indian British | 50.6 (46.3, 54.8) | 66.3 (61.5, 70.7) |
| Bangladeshi, Bangladeshi Scottish or Bangladeshi British | 47.1 (35.7, 58.8) | 69.6 (55.2, 80.9) |
| Chinese, Chinese Scottish or Chinese British | 62.7 (57.2, 67.9) | 95.1 (91.3, 97.3) |
| Other Asian | 18.3 (14.1, 23.5) | 32.2 (25.2, 40.1) |
| African | 39.6 (34.4, 45.1) | 69.3 (62.2, 75.6) |
| Caribbean or Black | 25.0 (18.2, 33.3) | 28.2 (20.6, 37.2) |
| Arab, Arab Scottish or Arab British | 11.6 (7.2, 18.3) | 57.7 (38.9, 74.5) |
| Other ethnic group | 16.0 (10.1, 24.4) | 6.1 (3.8, 9.7) |

B. Sensitivity and PPV for comparison between the Census and A&E using aggregated groups.

|  | **Sensitivity** | **PPV** |
| --- | --- | --- |
| **Ethnicity** | Estimate (95% CI) | Estimate (95% CI) |
| White | 86.9 (86.7, 87.0) | 99.7 (99.7, 99.7) |
| Mixed or multiple ethnic groups | 7.1 (4.4, 11.3) | 6.0 (3.8, 9.6) |
| Asian | 63.2 (61.3, 65.1) | 93.5 (92.3, 94.6) |
| African, Caribbean or Black | 46.7 (42.1, 51.4) | 70.6 (65.1, 75.5) |
| Other ethnic group | 20.5 (15.8, 26.2) | 16.3 (12.5, 21.0) |

C. Proportion of “refused” and “not known” codes for the comparison between the Census and A&E using aggregated groups.

| **Ethnicity** | **Refused**  **%** | **Not Known %** |
| --- | --- | --- |
| White | 2.8 | 10.2 |
| Mixed or multiple ethnic groups | 4.5 | 13.4 |
| Asian | 2.6 | 15.1 |
| African, Caribbean or Black | 5.3 | 18.5 |
| Other Ethnic Group | 5.7 | 10.5 |
| Total | 2.8 | 10.2 |

**Appendix 9**

| **Ethnic group** | ***HR (95% CI)*** | |
| --- | --- | --- |
|  | ***Census*** | ***PHS-EL*** |
| *Dis-aggregated groups* | | |
| White Scottish | Ref | Ref |
| White Other British | 0.58 (0.54 to 0.63) | 0.68 (0.64 to 0.71) |
| White Irish | 0.93 (0.80 to 1.07) | 0.75 (0.58 to 0.96) |
| White Gypsy/Traveller | 1.68 (1.03 to 2.74) | 0.73 (0.10 to 5.15) |
| White Polish | 0.87 (0.71 to 1.08) | 1.26 (0.99 to 1.60) |
| Other White | 0.62 (0.52 to 0.74) | 0.6 (0.52 to 0.69) |
| Mixed or multiple | 0.75 (0.49 to 1.15) | 0.97 (0.71 to 1.33) |
| Pakistani | 2.41 (2.14 to 2.72) | 2.37 (2.09 to 2.70) |
| Indian | 0.95 (0.74 to 1.23) | 1.06 (0.82 to 1.35) |
| Bangladeshi | 2.03 (1.20 to 3.44) | 1.45 (0.75 to 2.78) |
| Chinese | 0.57 (0.39 to 0.83) | 0.6 (0.41 to 0.89) |
| Other Asian | 1.33 (1.00 to 1.76) | 1.02 (0.74 to 1.43) |
| African | 1.7 (1.32 to 2.18) | 1.38 (1.02 to 1.85) |
| Caribbean or Black | 1.05 (0.60 to 1.85) | 1.86 (1.21 to 2.85) |
| Arab | 1.84 (1.25 to 2.70) | 1.31 (0.71 to 2.44) |
| Other Ethnicity | 1.26 (0.75 to 2.13) | 1.17 (0.85 to 1.61) |
| *Aggregated groups* | | |
| White | Ref | Ref |
| Mixed or multiple | 0.77 (0.50 to 1.19) | 1.01 (0.74 to 1.38) |
| Asian | 1.64 (1.49 to 1.80) | 1.59 (1.44 to 1.77) |
| African, Caribbean or Black | 1.6 (1.28 to 2.02) | 1.57 (1.23 to 2.01) |
| Other Ethnicity | 1.65 (1.21 to 2.25) | 1.25 (0.94 to 1.67) |

Hazard ratios (HR) and 95% confidence intervals (CI) for severe COVID-19 (hospitalisation and death) using dis-aggregated and aggregated ethnicity coding from the 2011 Scottish Census and from the Public Health Scotland ethnicity lookup (PHS-EL) dataset.
